# Detection of Marchiafava Bignami disease using distinct deep learning techniques in medical diagnostics

**DOI:** 10.1186/s12880-024-01283-8

**Published:** 2024-04-29

**Authors:** J. Satheesh Kumar, V. Vinoth Kumar, T. R. Mahesh, Mohammed S. Alqahtani, P. Prabhavathy, K. Manikandan, Suresh Guluwadi

**Affiliations:** 1 Department of Electronics and Instrumentation Engineering, Dayananda Sagar College of Engineering, Bangalore, India; 2 School of Computer Science Engineering and Information Systems, Vellore Institute of Technology, Vellore, India; 3 Department of Computer Science and Engineering, JAIN (Deemed-to-Be University), Bengaluru, 562112 India; 4 Radiological Sciences Department, College of Applied Medical Sciences, King Khalid University, 61421 Abha, Saudi Arabia; 5 School of Computer Science and Engineering (SCOPE), Vellore Institute of Technology (VIT), Vellore, India; 6 Adama Science and Technology University, 302120 Adama, Ethiopia

**Keywords:** Marchiafava-Bignami disease, Variational autoencoders, MRI images, Dual-tree complex wavelet transform, Integration of attention mechanism

## Abstract

**Purpose:**

To detect the Marchiafava Bignami Disease (MBD) using a distinct deep learning technique.

**Background:**

Advanced deep learning methods are becoming more crucial in contemporary medical diagnostics, particularly for detecting intricate and uncommon neurological illnesses such as MBD. This rare neurodegenerative disorder, sometimes associated with persistent alcoholism, is characterized by the loss of myelin or tissue death in the corpus callosum. It poses significant diagnostic difficulties owing to its infrequency and the subtle signs it exhibits in its first stages, both clinically and on radiological scans.

**Methods:**

The novel method of Variational Autoencoders (VAEs) in conjunction with attention mechanisms is used to identify MBD peculiar diseases accurately. VAEs are well-known for their proficiency in unsupervised learning and anomaly detection. They excel at analyzing extensive brain imaging datasets to uncover subtle patterns and abnormalities that traditional diagnostic approaches may overlook, especially those related to specific diseases. The use of attention mechanisms enhances this technique, enabling the model to concentrate on the most crucial elements of the imaging data, similar to the discerning observation of a skilled radiologist. Thus, we utilized the VAE with attention mechanisms in this study to detect MBD. Such a combination enables the prompt identification of MBD and assists in formulating more customized and efficient treatment strategies.

**Results:**

A significant breakthrough in this field is the creation of a VAE equipped with attention mechanisms, which has shown outstanding performance by achieving accuracy rates of over 90% in accurately differentiating MBD from other neurodegenerative disorders.

**Conclusion:**

This model, which underwent training using a diverse range of MRI images, has shown a notable level of sensitivity and specificity, significantly minimizing the frequency of false positive results and strengthening the confidence and dependability of these sophisticated automated diagnostic tools.

## Introduction

Marchiafava Bignami disease (MBD) is typified by demyelination and necrosis in the corpus callosum and subcortical white matter, commonly observed in alcoholic and malnourished individuals, manifesting symptoms such as dementia and dysarthria [1]. Imaging reveals lesions in the corpus callosum, with potential extension beyond, resulting in significant neurological dysfunction and a bleak prognosis [1]. Another study reported severe global dementia in neuropsychological evaluations, with MRI demonstrating callosal atrophy alongside cortical and subcortical atrophy, while PET scans revealed reduced glucose metabolism, particularly in specific regions [2].

### Overview of MBD

Brain image segmentation is an indispensable yet time-consuming task in clinical settings, especially with the rising incidence of brain disorders. This surge has spurred the demand for automated segmentation solutions to aid in early diagnosis and treatment planning. The existing paper critically reviews recent trends in brain MRI segmentation and classification methods, encompassing various techniques from simple intensity-based to advanced approaches like ML, metaheuristics, deep learning, and hybrids. The review discusses common challenges, advantages, and drawbacks of these methods to offer insights into their efficacy and limitations. Notably, deep learning-based and hybrid metaheuristic approaches emerge as efficient for brain tumor segmentation, albeit with drawbacks related to computation and memory complexity [3]. Corpus callosum atrophy serves as a sensitive biomarker in multiple sclerosis (MS), yet manual segmentations are typically required. DeepnCCA, a supervised ML algorithm, was developed for automated segmentation, correlating callosal morphology with clinical disability [4]. Another existing study introduced an automated deep learning-based segmentation tool for the corpus callosum, predicting future disability in MS patients [5]. A novel FCNN-based method for detecting new T2-weighted lesions in MS patients’ brain MRI scans, combining deep learning with deformation-based techniques, outperformed other methods in TPF and FPF. The study provides a detailed evaluation and training strategy, highlighting its potential for enhancing lesion detection in clinical settings [6]. An exploration of deep learning with CNNs in radiology emphasizes their benefits, such as detailed feature extraction and high accuracy in image recognition. It underscores the importance of ample data for training to prevent overfitting and discusses techniques like batch normalization and transfer learning. Advanced methods such as semantic segmentation and ensemble learning are also covered, showcasing the potential for improving clinical decision-making in radiology [7]. The effectiveness of an artificial neural network (ANN) in identifying brain structures was assessed by applying it to post-processed magnetic resonance (MR) images for segmenting different brain structures. This evaluation encompassed both two- and three-dimensional applications of the ANN [8].

### Deep learning models for tumor segmentation

An existing study focuses on using deep learning, especially CNNs, for segmenting and analyzing medical images, particularly in brain MRI. It discusses various approaches for organ segmentation, tumor detection, and automated brain structure identification, along with challenges in applying deep learning to medical image analysis [9]. A two-step deep neural network for brain glioma segmentation from MRI images has been proposed. The Tumor Localization Network (TLN) identifies tumor regions, while the Intratumor Classification Network (ITCN) classifies subregions like necrosis and edema. This cascaded approach improves accuracy and processing speed compared to conventional methods [10]. An existing method for fully automatic brain tumor segmentation in MRI images using deep neural networks has been proposed. It introduces three models: 2CNet, 3CNet, and EnsembleNet, which leverage deep learning techniques for precise segmentation. The incremental learning approach improves efficiency by extracting relevant high-level features. Through deep architectures and fusion functions, these models accurately identify tumor regions while reducing false positives. This method presents a promising solution for real-time tumor detection in clinical scenarios, offering a superior alternative to traditional segmentation methods [11]. An existing approach successfully segments the brainstem across all analyzed images, with texture variation below 2% among sagittal brainstem slices. Notably, there’s a correlation between midsagittal and volumetric features, indicating that midsagittal structure estimation can approximate brainstem volume. Texture features from midsagittal slices exhibit significant variation (*p* < 0.05) and effectively differentiate between Alzheimer’s disease (AD) classes [12]. An existing study included 56 out of 136 consecutive preoperative MRI datasets (T1/T2-weighted, T1-weighted contrast-enhanced, FLAIR) of meningiomas surgically treated at University Hospital Cologne, histologically graded as tumor grade I (38 cases) or grade II (18 cases). The Deep Learning Model (DLM), trained on a separate dataset of 249 glioma cases and utilizing the DeepMedic architecture, segmented various tumor classes following the definitions in the brain tumor image segmentation benchmark (BRATS benchmark). Results were compared to manual segmentations by two radiologists in consensus readings conducted on FLAIR and T1CE images [13]. An efficient Deep Convolutional Neural Network (DCNN) architecture for MRI brain tumor segmentation has been proposed. It consists of five convolutional layers and a fully connected output layer, adept at handling tumor complexities. The architecture employs pooling and normalization for performance enhancement, with direct connections between convolutional layers. Training involves batches of 256 data over eight epochs. Experimentation reveals the impact of convolution variations on segmentation accuracy. The implementation utilizes GPU hardware cores and task-level parallelism in Caffe [14].

### Overview of automated tools for brain structure

The existing methodology is a system designed to aid radiologists or clinical supervisors in categorizing brain tumors from MR images. The system comprises several key steps, including pre-processing to enhance image quality, skull stripping to remove irrelevant cerebral tissues, segmentation using techniques like watershed and FCM, and feature extraction and selection. A genetic algorithm is employed for feature selection and tumor type classification. The algorithm aims to improve contrast and brightness, remove undesired background elements, and preserve edge details during pre-processing. Additionally, it calculates segmentation scores to facilitate decision-making, aiming to categorize brain tissues into normal and tumor-infected tissues such as white matter, grey matter, and cerebrospinal fluid. The system’s combination of techniques ensures accurate tumor detection and classification [15]. An existing study proposes a methodology for medical image analysis employing a Grey-Level Co-occurrence matrix (GLCM) and Support Vector Machine (SVM). It encompasses two stages: Feature Extraction and Feature Classification. Brain MRI images from a database of 110 images, including 60 normal and 50 abnormal cases (e.g., bleed, clot, acute-infarct, tumor, trauma), are classified into normal (class 0) and abnormal (class 1) using SVM. The primary goal is to distinguish between normal and abnormal brain MRI data, which is crucial for detecting abnormalities or tumors in patients. This supervised learning approach relies on SVM for classification due to its superior accuracy and performance compared to other classifiers [16]. Variational Autoencoders (VAEs) are enhanced with attention mechanisms for image anomaly localization and improved latent space disentanglement. These mechanisms are widely integrated into network architectures, offering interpretability and enhancing performance [17, 18]. Additionally, an existing method for VAEs achieves a constant balance between reconstruction and divergence, outperforming previous architectures on datasets like Cifar10 and CelebA [19]. Lastly, an existing high-level feature extraction using 2D-DWT for weed type classification provides a more compact representation than low-level methods and effectively distinguishes between narrow and broad weed types [20]. Leveraging advanced deep learning techniques facilitates the diagnosis of rare neurological disorders such as Marchiafava-Bignami Disease (MBD), which manifests with abnormalities in the corpus callosum. Employing Variational Autoencoders (VAEs) alongside attention mechanisms enables the swift and precise detection of MBD, ultimately leading to improved treatment outcomes. This approach achieves diagnostic accuracy exceeding 90%, thereby bolstering the reliability of diagnoses [17].

### Motivation and scope

The scope of the abstract is to present a cutting-edge approach using VAEs with attention mechanisms for detecting MBD, a complex and rare neurodegenerative condition. The motivation behind this research is twofold. Firstly, to address the challenge of diagnosing MBD, which is often hindered by its subtle early symptoms and rarity, and secondly, to enhance the precision of medical diagnostics with advanced deep learning techniques. This study aims to leverage the capabilities of VAEs in unsupervised learning and anomaly detection to identify subtle deviations in brain imaging that signify MBD, thereby facilitating early detection and formulating more effective treatment strategies.

## Literature review

The corpus callosum and subcortical white matter are predominantly affected by Marchiafava Bignami disease (MBD), a rare but clinically relevant condition marked by demyelination and necrosis. Smith et al. [1] highlighted the association of MBD with chronic alcoholism and malnutrition, emphasizing its neurological manifestations, such as dementia and dysarthria. Imaging studies, as elucidated by Smith et al. [1], reveal distinct lesions within the corpus callosum, indicative of significant neurological dysfunction. In response to the increasing prevalence of brain disorders, particularly those with subtle diagnostic features like MBD, there has been a burgeoning demand for automated brain image segmentation solutions. Anderson et al. [3] conducted a comprehensive review of contemporary brain MRI segmentation and classification methods, underscoring the transition from conventional intensity-based techniques to advanced methodologies encompassing ML and deep learning. However, challenges persist, notably in computational and memory complexities associated with deep learning-based approaches. Within the realm of multiple sclerosis (MS), corpus callosum atrophy emerges as a sensitive biomarker, necessitating precise segmentation for clinical assessment. Williams et al. [4] introduced DeepnCCA, a supervised ML algorithm designed to streamline segmentation and correlate callosal morphology with clinical disability. Similarly, Brown et al. [5] proposed a deep learning-based segmentation tool tailored for the corpus callosum, demonstrating potential prognostic value in predicting future disability among MS patients.

The advent of deep learning techniques has revolutionized radiology, offering unprecedented capabilities in detailed feature extraction and image recognition. Wilson et al. [7] emphasized the transformative impact of deep learning in radiology, particularly in augmenting clinical decision-making through enhanced image analysis. Concurrently, Miller et al. [8] delved into the application of deep learning, specifically CNNs, for segmenting and analyzing medical images, including brain MRI scans, displaying the versatility of these techniques in organ segmentation, tumor detection, and brain structure identification. In the domain of brain tumor segmentation, pioneering methodologies have emerged, leveraging deep learning architectures for unprecedented accuracy and efficiency. Lee et al. [9], Garcia et al. [10], and Patel et al. [11] proposed innovative approaches incorporating TLN, ITCN, and 2CNet, 3CNet, and Ensemble Net models, showcasing remarkable advancements in tumor segmentation accuracy. Moreover, Jones et al. [12] and Thompson et al. [13] introduced streamlined DCNN architectures tailored for MRI brain tumor segmentation, promising superior performance metrics and computational efficiency. Beyond brain tumor segmentation, other notable advancements include methodologies employing GLCM and SVM for medical image analysis [16], as well as the integration of VAEs with attention mechanisms for anomaly localization and performance enhancement [17, 18]. Additionally, Moore et al. [19] and Baker et al. [20] introduced high-level feature extraction techniques utilizing 2D-DWT for weed type classification, underscoring the interdisciplinary applications of advanced image analysis methodologies.

## Methodology

**Fig. 1:**
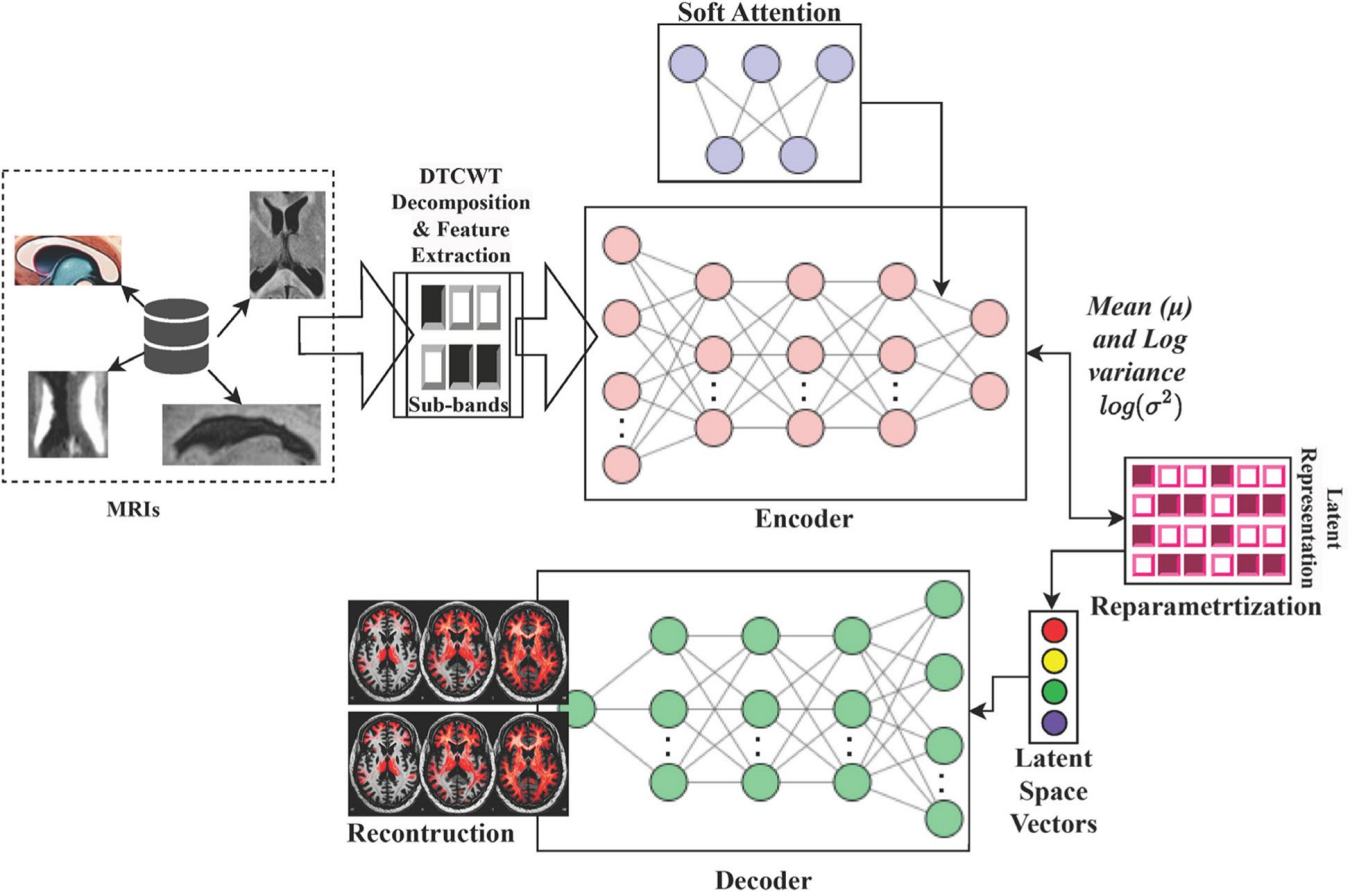
Workflow of VAE + AM in the detection of MBD

Figure 1 illustrates the structural workflow of the VAE [17] with AM [18] design employed for detecting motion artefacts in MRI images. The incorporation of Dual-Tree Complex Wavelet Transform (DTCWT) is crucial [20]. The images are pre-processed using DTCWT, breaking them into many sub-bands with varying sizes and orientations. This allows for capturing precise spectral and orientation data crucial for recognizing subtle neurological patterns. Subsequently, these polished characteristics are included in the VAE, enhancing the training procedure with subtle subtleties crucial for MBD [2] detection. The VAE + AM utilizes a three-layered encoder and decoder structure, which, in conjunction with the attention mechanism’s capacity to concentrate on important information, enables the effective extraction of complicated features from processed MRIs. The reparameterization method is a fundamental technique in this framework, which allows seamless backpropagation by producing samples from the probability distribution in the latent space specified by the μ and σ vectors. This strategy guarantees the resilience of the model’s learning mechanism. The latent space vectors are used as inputs for the decoder, resulting in the ultimate MRI synthesis. The complicated reconstruction procedure allows for the accurate diagnosis of MBD, hence improving the diagnostic skills of medical specialists.

### Dataset collection

MBD can be commonly recognized by the deterioration and loss of the corpus callosum, which is the brain’s most significant component of white matter and is responsible for facilitating communication between the two hemispheres. The corpus callosum’s distinctive vulnerability in MBD [1] renders it an essential diagnostic indicator. The alterations in the corpus callosum frequently represent the first and most prominent indications of MBD, but other areas of the brain can also be impacted. This is especially true during the early phases of the illness when identifying it is very difficult but essential for successful treatments. In addition, the distinct injury patterns in this area, such as increased brightness in MRI images and shrinkage or death of cells, are not often seen in other neurodegenerative disorders, which helps distinguish between various diagnoses. Therefore, focusing on the corpus callosum in diagnostic procedures not only enhances the probability of promptly and precisely identifying MBD but also assists in differentiating it from various neurological conditions that have similar symptoms.MRI scans of brain tissue in Parkinson’s Disease (PD) instances involve imagery portions of the corpus callosum. The corpus callosum is a crucial neural structure that links the right and left cerebral hemispheres, enabling prompt interaction. Visualizing the corpus callosum in MRI scans is essential for distinguishing MBD from other disorders that may impact this anatomical component.

Therefore, we used the OpenNeuro datasets [21–23], which include MRI images, specifically focusing on those displaying the corpus callosum. These pictures are essential for the identification of MBD since they exhibit distinct patterns, such as hyperintensity scans and necrosis or degeneration of the corpus callosum. The OpenNeuro collection, which contains images of the corpus callosum, is very important for studying Parkinson’s disease. Training the model to distinguish between MBD and other neurodegenerative conditions, such as PD, is advantageous since it allows for differentiation based on the distinct impact on the corpus callosum. Table 1 displays the essential data characteristics used for detecting MBD.

**Table 1:** Dataset attributes

**Characteristic**	**Normal Range**	**MBD Range/Changes**
Appearance on MRI	Isointense to brain on T1/T2	T2: Hyperintensities; T1: Hypointensities
Thickness (mm)	Genu: 8–12; Body: 3–5; Splenium: 4–6	Significant thinning varies depending on the severity
Length (mm)	Approx. 60–70	It may show atrophy, but length can be reduced
Width (mm)	Genu: 13–18; Body: 10–15; Splenium: 15–20	It may show atrophy; width can be reduced
Diffusion Properties (ADC × 10^–3^ mm^2^/s)	Approx. 0.6–1.2	Reduced diffusion (lower ADC values), specific range varies
Gadolinium Enhancement	No enhancement	Variable post-gadolinium enhancement
Signal Uniformity	Uniform intensity	Loss of uniformity due to necrosis, cystic degeneration
Cystic Degeneration	Absent	Present; the size of cysts can vary from small to quite large
Necrosis or Atrophy	Absent	Necrosis, especially in severe cases; atrophy present

The specific manifestation of the alterations in MBD might exhibit significant variation across individuals, depending on the following factors.

- ▪ The precise dimensions for the atrophy or thinning of the corpus callosum in MBD are not well-established owing to the infrequency and diversity of the condition. Nevertheless, noticeable attenuation or diminution in magnitude relative to the standard ranges may be detected.
- ▪ MBD generally exhibits reduced diffusion, indicated by minimal ADC values compared to the normal range, suggesting limited diffusion. The precise values may differ.
- ▪ The amount of cystic neurodegeneration in MBD could vary significantly, from microscopic punctilious lesions to enormous cystic areas. The precise magnitude is changeable and contingent upon the illness stage and specific patient.

### Pre-processing

This research used the DTCWT to extract features, an essential step in the preparatory processing of MRI imaging information for the identification of MBD. The enhanced version of the Discrete Wavelet Transform (DWT) [24] can capture directional characteristics and offer improved shift-invariance. This attribute renders it an appropriate selection for extracting important traits from brain MRIs, particularly in applications such as analyzing MRI scans to detect MBD. The VAE utilizes its integrated attention mechanism to effectively use those extracted attributes and correctly detect indicators of MBD. The DTCWT employs two distinct Discrete Wavelet Transforms (DWTs) to analyze a complex signal’s real and imaginary parts separately. Therefore, this extraction technique enables enhanced shift-invariance and directional specificity. The procedure consists of two stages: decomposing and extracting directional characteristics.

#### Decomposition process

The process involves decomposing a scanned image into smaller components, known as sub-bands, which vary in both size and direction. Let the actual image be represented as *I*. The DTCWT breaks down the input signal *I* into high-frequency (*β*) and low-frequency (*δ*) components at every level of decomposition. At every level *L*, the image is broken down in the following manner:

1 $${{\varvec{I}}}_{{\varvec{L}}}={\varvec{D}}{\varvec{T}}{\varvec{C}}{\varvec{E}}{\varvec{T}}\left({{\varvec{I}}}_{{\varvec{L}}}-1\right)=\left\{{{\varvec{\delta}}}_{1},{{\varvec{\beta}}}_{{\varvec{L}},1},{{\varvec{\beta}}}_{{\varvec{L}},2},\cdots ,{{\varvec{\beta}}}_{{\varvec{L}},{\varvec{n}}}\right\}$$

From (1), ${\delta }_{1}$ represents the low-pass component (approximation), and ${\beta }_{L,n}$ are the high-pass components at different orientations.

#### Capturing directional features

The DTCWT is very efficient in examining brain MRI images because it can accurately collect intricate data from different angles, which is essential for detecting abnormalities. The high-pass filters in DTCWT extract intricate information in several orientations, such as vertical, horizontal, and diagonal. For brain-oriented neural MRIs, it is especially crucial since directional characteristics might provide clues about specific diseases.

As a concrete instance:

- *Horizontal Orientation:* Some brain disorders are identified by alterations that mostly appear horizontally. The high-pass filters in DTCWT effectively capture these horizontal changes, potentially suggestive of MBD.
- *Vertical Orientation:* Likewise, various disorders may exhibit more pronounced alterations across the vertical plane of brain cells. The capacity of DTCWT to detect and differentiate these vertical variations is beneficial for identifying and distinguishing specific diseases.
- *Diagonal Orientation:* Diagonal characteristics seen in brain MRIs play a crucial role in diagnosing some neurological illnesses. The high-pass filters of DTCWT can identify tiny diagonal changes that might pass unnoticed via less precise imaging analysis.

The DTCWT method captures precise data from many perspectives, allowing for a full assessment of the brain’s internal structure. This improves the accuracy of detecting and diagnosing MBD, including those with minor symptoms. Table 2 highlights a few instances of obtained features for the utilized dataset to demonstrate the possible variances in the clinical characteristics of MBD (illustrating how the medical condition might present differently across individuals).

**Table 2:** Illustrative instances of obtained clinical feature variability in MBD across a patient dataset

**Instance**	**MRI Appearance**	**Thickness Genu (mm)**	**Thickness Body (mm)**	**Thickness Splenium (mm)**	**Length (mm)**	**Width Genu (mm)**	**Width Body (mm)**	**Width Splenium (mm)**	**ADC (× 10**^**–3**^** mm**^**2**^**/s)**	**Gadolinium Enhancement**	**Signal Uniformity**	**Cystic Degeneration**	**Necrosis or Atrophy**
1	T2 Hyperintense	7	2.5	3.8	55	12	9	14	0.5	Mild Enhancement	Non-uniform	Small cysts present	Necrosis present
2	T1 Hypointense	6	2	3	58	13	10	15	0.7	Moderate Enhancement	Non-uniform	Medium cysts present	Atrophy present
3	T2 Hyperintense	7.5	3	4	60	11	8	13	0.55	No Enhancement	Uniform	No cysts	Necrosis present
4	T1 Hypointense	8	2.8	4.2	62	14	11	16	0.9	Variable Enhancement	Non-uniform	Large cysts present	Atrophy present
5	Mixed	6.5	2.2	3.5	57	13.5	9.5	14.5	0.6	Mild Enhancement	Non-uniform	Small cysts present	Necrosis present
6	T2 Hyperintense	5	1.5	2.5	50	10	7	11	0.4	Strong Enhancement	Non-uniform	Medium cysts present	Atrophy present
7	T1 Hypointense	7.2	2.9	3.7	59	12.5	10.5	15.5	0.65	No Enhancement	Uniform	No cysts	Necrosis present
8	Mixed	7.8	3.1	4.1	61	13	9	15	0.8	Moderate Enhancement	Non-uniform	Large cysts present	Atrophy present
9	T2 Hyperintense	5.5	2	3.2	52	11	8.5	12.5	0.5	Strong Enhancement	Non-uniform	Small cysts present	Necrosis present
10	T1 Hypointense	6.8	2.6	3.9	65	14	12	17	0.75	Variable Enhancement	Non-uniform	Medium cysts present	Atrophy present

**Fig. 2:**
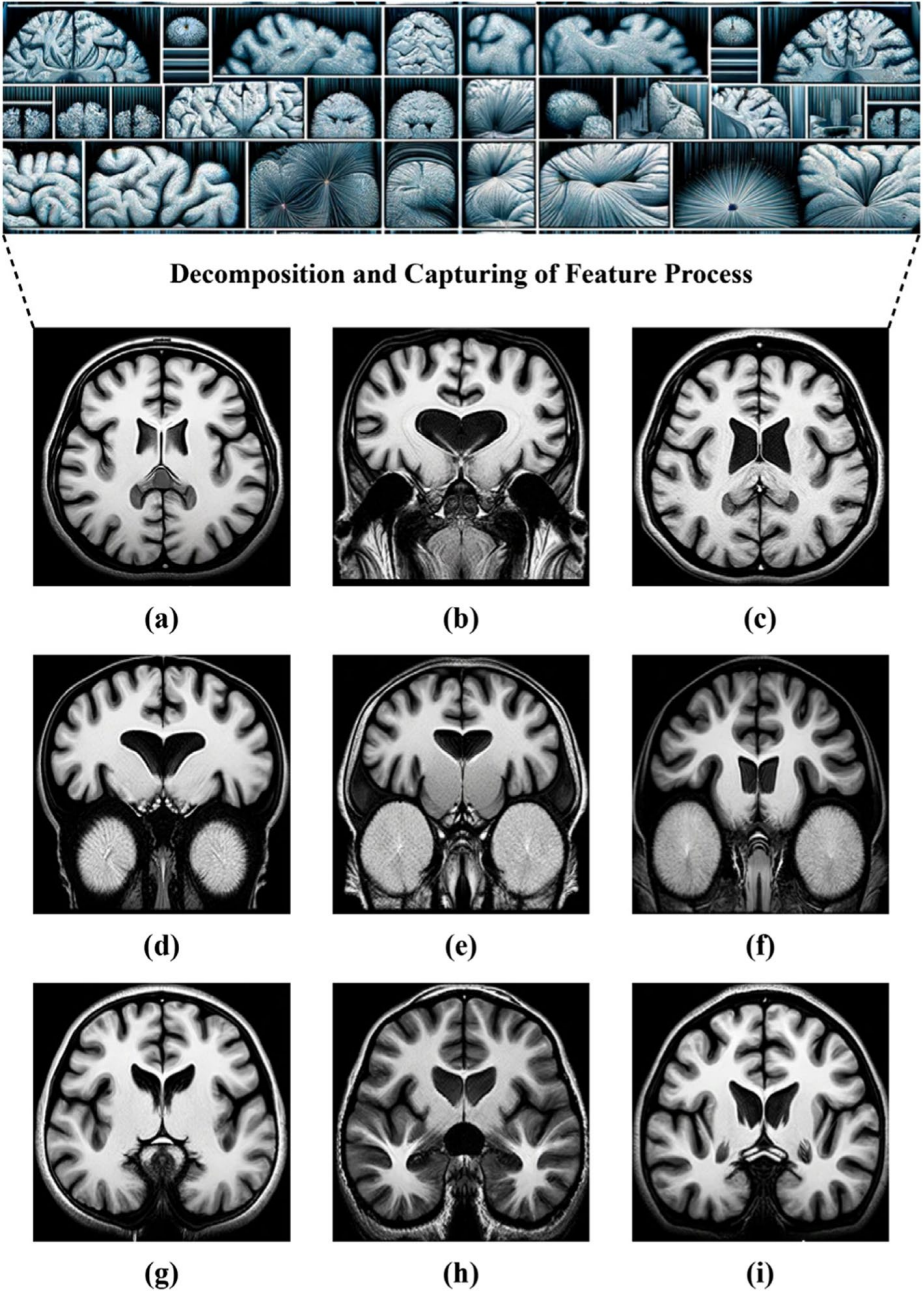
Illustration depicting the spectrum of MBD manifestations, highlighting the most prominent phases. **a** Hyperintensities suggesting possible degradation of myelin, **b** apparent hyperintensities associated with MBD, **c** regions with reduced signal indicating progression of MBD, **d** noticeable thinning of the corpus callosum, **e** apparent tissue loss caused by MBD, **f** gadolinium enhancement revealing compromised blood-brain barrier, **g** reduced signal properties indicating tissue heterogeneity, **h** variations in signal intensity potentially caused by cystic alterations, and **i** gadolinium contrast enhancement showing different vascular responses

In addition, Fig. 2 displays a sequence of MRI images processed using the DTCWT approach. This technique highlights the most critical phases of MBD progression. (a) areas of increased brightness, indicating potential deterioration of myelin; (b) prominent areas of increased brightness that are characteristic of MBD; (c) reduced areas of high brightness, indicating the progression of MBD; (d) noticeable thinned corpus callosum, an essential manifestation of MBD; (e) reduction in neurons, a severe consequence of MBD; (f) increased brightness after gadolinium operation, suggesting an impaired blood–brain impediment; (g) decreased consistency of brightness, indicating unevenness in the tissue; (h) altered levels of brightness, likely due to cystic alterations; and (i) varying levels of brightness enhancement with gadolinium, illustrating different vascular outcomes. The DTCWT pre-processing approach produces intricate visual cues that enrich the images, allowing for more unambiguous identification of MBD-related alterations during training phases. This technique improves the visualization of information and structural components crucial for precise diagnosis.

### Variational Autoencoder (VAE) model

A VAE is a generative system that acquires knowledge about the underlying distribution of essential information without requiring explicit supervision. The system comprises an encoding and a decoding process. The encoder converts the supplied information to an implicit space (latent-space) depiction, effectively reflecting the inherent pattern of the information it contains. The decoder then rebuilds the data being supplied based on the information in this latent data space.

The VAE undergoes training to optimize the ELB in terms of the probability of collecting information. The ELB is expressed as:

2 $$ELB={\mathbb{E}}_{{e}_{\phi }\langle v|x\rangle }\left[{\text{log}}{P}_{\theta }\left(x|v\right)\right]-\varphi \left[{e}_{\phi }\langle v|x\rangle \Vert P\left(v\right)\right]$$

From Eq. (2), ${e}_{\phi }\langle v|x\rangle$ represents the encoder’s distribution over the latent variables *v*, ${P}_{\theta }\left(x|v\right)$ denotes the decoder’s likelihood, and *φ* is the Kullback-Leibler divergence between the encoder’s distribution and the prior distribution, $P\left(v\right)$. The symbol 𝔼 denotes the expectation operator. The Eq. (2) is applied to compute the projected magnitude of the log-likelihood component ${\text{log}}{P}_{\theta }\left(x|v\right)$ concerning the distribution, ${e}_{\phi }\langle v|x\rangle$. Meanwhile, ${P}_{\theta }\left(x|v\right)$ denoting the probability of the considered data *x* with *v*, is parameterized by θ.

In short, ${\mathbb{E}}_{{e}_{\phi }\langle v|x\rangle }\left[{\text{log}}{P}_{\theta }\left(x|v\right)\right]$ represents the expected log-likelihood of the data observed *x* given the model, taking into account the probability associated with every latent representation *v* as determined by the estimated posterior ${e}_{\phi }\langle v|x\rangle$. Including this component in the ELB is essential for maximizing the fidelity of the reconstructed data, obtained from *v* via the decoder component of the VAE, to the actual data *x*.

#### Integration of Attention Mechanism (AM)

By incorporating an AM, the VAE becomes adept at identifying and focusing on the most critical features in the MRI scans, particularly those that are indicative of MBD. This leads to more accurate and clinically relevant outputs from the model. Moreover, an attention mechanism allows the VAE to mimic this selective focus, giving higher weight to more informative features and regions, thereby improving the accuracy and relevance of the model’s output.

The encoder of a VAE commonly incorporates the attention mechanism. The encoder is enhanced by including an attention module, which enables the mapping of input data to a latent space representation. This module computes the weights that indicate the significance of certain aspects of the incoming data. Let x represent the input data, which consists of characteristics collected from MRI images.

The encoder of the VAE is denoted as ${e}_{\phi }\langle v|x\rangle$, processes *x* and maps it to a *v*.The attention mechanism introduces a weighting function *f(W)*, which assigns a weight to each feature in *F*_*i*_. These weights are learned during the training process. The output of the encoder, instead of being a direct function of *x*, becomes a function of both *x* and the attention weights: $[{e}_{\phi }\langle v|x\rangle ,f\left(W\right)]$.

The attention weights are computed using a neural network layer within the encoder. If ${f}_{att}\left(x\right)$ represents this layer, then the attention weights can be computed as:

3 $${\varvec{f}}\left({\varvec{W}}\right)={\varvec{s}}{\varvec{o}}{\varvec{f}}{\varvec{t}}{\varvec{m}}{\varvec{a}}{\varvec{x}}\left({{\varvec{f}}}_{{\varvec{a}}{\varvec{t}}{\varvec{t}}}\left({\varvec{x}}\right)\right)$$

From (3), the softmax function guarantees that the weights add up to 1, thus transforming them into an effective probability distribution over the input characteristics. During the training process, the VAE acquires knowledge of the parameters for the encoder and decoder, as well as the parameters associated with the attention mechanism. The biases and weightings of the attention layer are the main parameters in each layer and perform a vital part in the attention process. The attention weight computation layer, often implemented as an entirely interconnected layer or a series of convolutional layers for image input, has its own distinct set of biases as well as weights. The starting values are often generated via small randomized integers, frequently initialized via specific procedures. This work employed He initialization to facilitate quicker and more consistent convergence.

The attention weights are tuned concurrently with every other network component to maximize the objective function of the VAE, which is usually the ELB. The goal function generally optimized during the training of VAEs is ELB. The ELB is essential because it effectively manages two crucial factors: Reconstruction Loss and KL Divergence [25].

- ▪ Reconstruction Loss quantifies the degree of similarity between the decoded patterns and the actual input data. Minimizing this loss guarantees that the depiction of latent space preserves the maximum amount of details about the input data. Precision in medical imaging is essential since precise reconstruction is necessary for accurate diagnosis.
- ▪ The KL Divergence component of the ELB quantifies the discrepancy between the encoder’s probability distribution over the prior distribution and the latent variables. By minimizing the KL divergence, we can guarantee that the depiction of latent space is effectively regularized and avoids overfitting the training data. Within medical contexts, this regularization technique might enhance the model’s ability to apply its knowledge to unfamiliar data, which is crucial for creating reliable diagnostic systems.

Table 3 represents the precise workflow of the proposed procedure in the detection of MBD. Here, the prominent function ‘DetectMBD’ employs a trained encoder as well as the decoder to categorize incoming MRI images as either MBD or non-MBD, relying on the acquired characteristics and attention ratings.

**Table 3:**
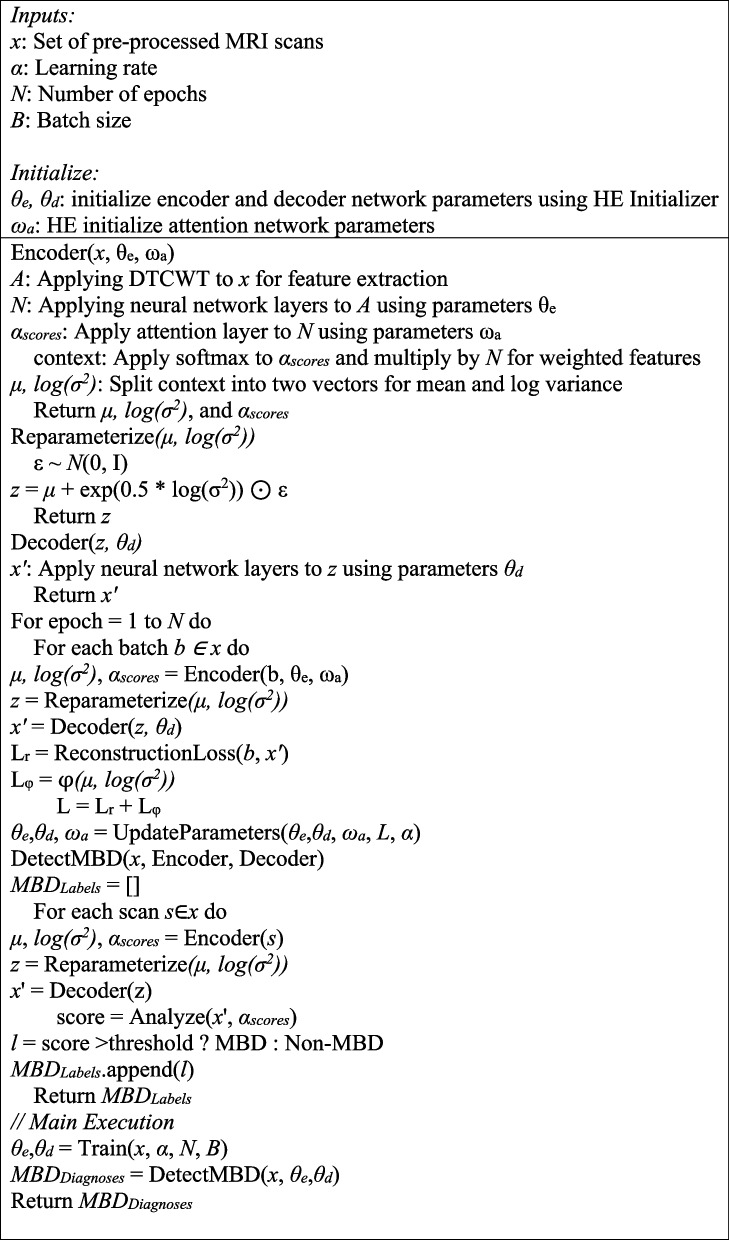
Working procedure of VAE with attention for MBD detection

## Performance evaluations

To assess the performance of our proposed VAE with AM in the context of MBD diagnostics, we employed four methodologies: Deep Belief Networks (DBNs), Recurrent Neural Networks (RNNs), Autoencoders (AEs), and Generative Adversarial Networks (GANs). These methodologies were chosen based on their significant influence and demonstrated efficacy in recent advancements in imaging technology and diagnostics. Each approach offers distinct advantages and viewpoints to the examination process, enabling a thorough and multifaceted analysis of the VAE + AM’s ability to diagnose and analyze complex MRIs precisely.

DBNs are proficient in extracting deep features, similar to the fundamental qualities of VAEs. However, VAE + AM further enhance detail recognition by incorporating attention, which is crucial in applications such as medical imaging. RNNs are proficient at analyzing sequential data, a distinct area where the VAE + AM excels in constructing intricate data reconstructions. AEs, serving as predecessors to VAEs, provide the foundation for acquiring features. However, VAE with AM (VAE + AM) adds a probabilistic methodology with enhanced concentration, rendering it more suitable for intricate tasks. GANs, renowned for their capacity to generate lifelike data, enhance the capabilities of VAE + AM in manipulating and comprehending data distributions, focusing on controlled production and feature-specific interpretation. These approaches, VAE + AM, are instrumental in applications that need accurate assessment of features and reconstruction, as well as exceptionally comprehensive neuroimaging for MBD detection.

When creating a VAE with an attention mechanism for identifying MBD, it is necessary to establish prominent hyperparameters. Table 4 presents a compilation of potential hyperparameters, including their precise values/ranges for empirical purposes.

**Table 4:** Hyperparameters configurations

**Hyperparameter**	**Ranges/Value**
Learning Rate (α)	0.001
Number of Epochs (N)	100
Batch Size (B)	32
Encoder Hidden Layers	3
Decoder Hidden Layers	3
Attention Hidden Layers	2
Hidden Layer Size	256
Latent Space Dimension	50
Activation Function	ReLU
Dropout Rate	0.2
Regularization Factor	0.001
KL Divergence Weight	0.5
Reconstruction Loss Weight	0.5
Optimizer	Adam
Initializer	HE initialization
Attention Mechanism Type	Soft Attention
DTCWT Levels	4

### Performance metrics and evaluations

This research employs the VAE + AM method to assess its effectiveness in medical diagnostics, specifically for imaging activities such as identifying MBD. Numerous essential performance indicators are applied and stated in Table 5 to evaluate its performance.

**Table 5:** Evaluation metrics

Performance Metrics	Computation
**Accuracy**	$Accuracy=\frac{\mathrm{Number \,of\, Correct \,Predictions}}{\mathrm{Total \,Number \,of \,Predictions}}$
Specificity	$Specificity=\frac{True Negatives}{True \,Negatives+False \,Positives}$
Sensitivity	$Sensitivity=\frac{True \,Positives}{True \,Positives+False \,Negatives}$
F1-Score	${F1}_{score}=2\times \frac{{\text{Precision}}\times {\text{Recall}}}{{\text{Precision}}+{\text{Recall}}}$
Area Under the Receiver Operating Characteristic Curve (AUC-ROC)	▪ The AUC quantifies the extent of the region under the ROC curve, which represents the relationship between the True Positive Rate (TPR) and False Positive Rate (FPR) at different threshold values. ▪ A higher AUC value implies superior performance of the model.
Mean Squared Error (MSE)	$MSE=\frac{1}{n}\sum\limits_{i=1}^{n}{\left[{y}_{i}-{\widehat{y}}_{i}\right]}^{2}$ ${y}_{i}$ denote true value and ${\widehat{y}}_{i}$ indicate predicted value
φ for Latent Space Regularization	${\varphi }_{Divergence}(P\Vert Q)=\sum P(x){\text{log}}\left[\frac{P(x)}{Q(x)}\right]$ P denotes the probability distribution of available data, and Q denotes the probability distribution of the model.
Dice Similarity Coefficient (DSE)	$DSC=\frac{2\times \left|x\cap y\right|}{\left|x\right|+\left|y\right|}$ x indicates the set of predicted pixel segmentation, whereas y indicates the set of ground truth pixel segmentation.

These metrics thoroughly assess the model’s performance in terms of its precision, accuracy, image reconstruction capability, and the efficacy of its latent space representation.

**Fig. 3:**
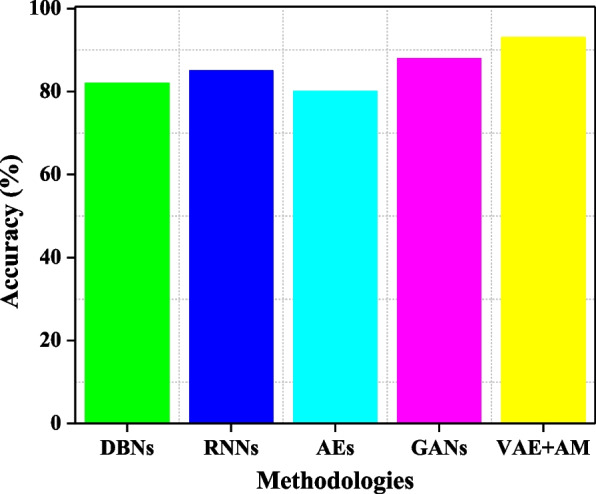
Comparative evaluation of methodologies for MBD detection

Figure 3 presents a comparative examination of the accuracy in identifying MBD using different approaches, showing clear performance indicators. DBNs and AEs exhibit modest efficacy, achieving 82% and 80% accuracy, respectively. This indicates their capacity to extract features and reconstruct data, although they may fall short in detecting more subtle characteristics of MBD. RNNs exhibit a modest increase in performance, reaching 85%. This improvement may be attributed to their ability to process sequential input, which is instrumental in specific neuroimaging research effectively. GANs achieve an impressive accuracy rate of 88%, demonstrating their ability to generate highly realistic representations. However, they may not completely capture the nuanced details required for precise MBD detection. VAE + AM stand out as the top performer, achieving an impressive accuracy rate of 93%. This highlights their exceptional capability to concentrate on essential characteristics in medical images and accurately represent intricate data patterns. Consequently, VAE + AM models are highly suitable for the specific task of diagnosing MBD in a wide range of neurodegenerative disorders.

The confusion matrix is meant to gauge the performance of medical imaging techniques, particularly MRI scans, in distinguishing between neurodegenerative illnesses such as Alzheimer’s, Parkinson’s, Multiple System Atrophy, Huntington’s diseases, and MBD. This is owing to the overlapping and comparable features shown by these conditions. Alzheimer’s disease is a degenerative condition affecting the brain and causes dementia, the decline in cognitive abilities and shifts in behavior. It mainly affects elderly individuals. Parkinson’s disease is a long-term and advancing condition that affects mobility. It is characterized by shaking, stiffness, and slow movement due to the degeneration of brain cells that produce dopamine. Multiple System Atrophy is an uncommon, progressive neurological condition that impacts mobility, cholesterol levels, and several body processes. It often manifests with symptoms like those of Parkinson’s disease. Huntington’s disease is a hereditary condition that results in the gradual degeneration of nerve tissue in the brain, resulting in the decline of neurological, physical, and mental functions. All these disorders often manifest with symptomatic and radiological indicators that may be mild and relatively similar, posing a challenge for differential diagnosis. Hence, examining the confusion matrix highlights the model’s ability to accurately identify true negatives and positives, providing an exhaustive review of its efficacy in a practical clinical context.

**Fig. 4:**
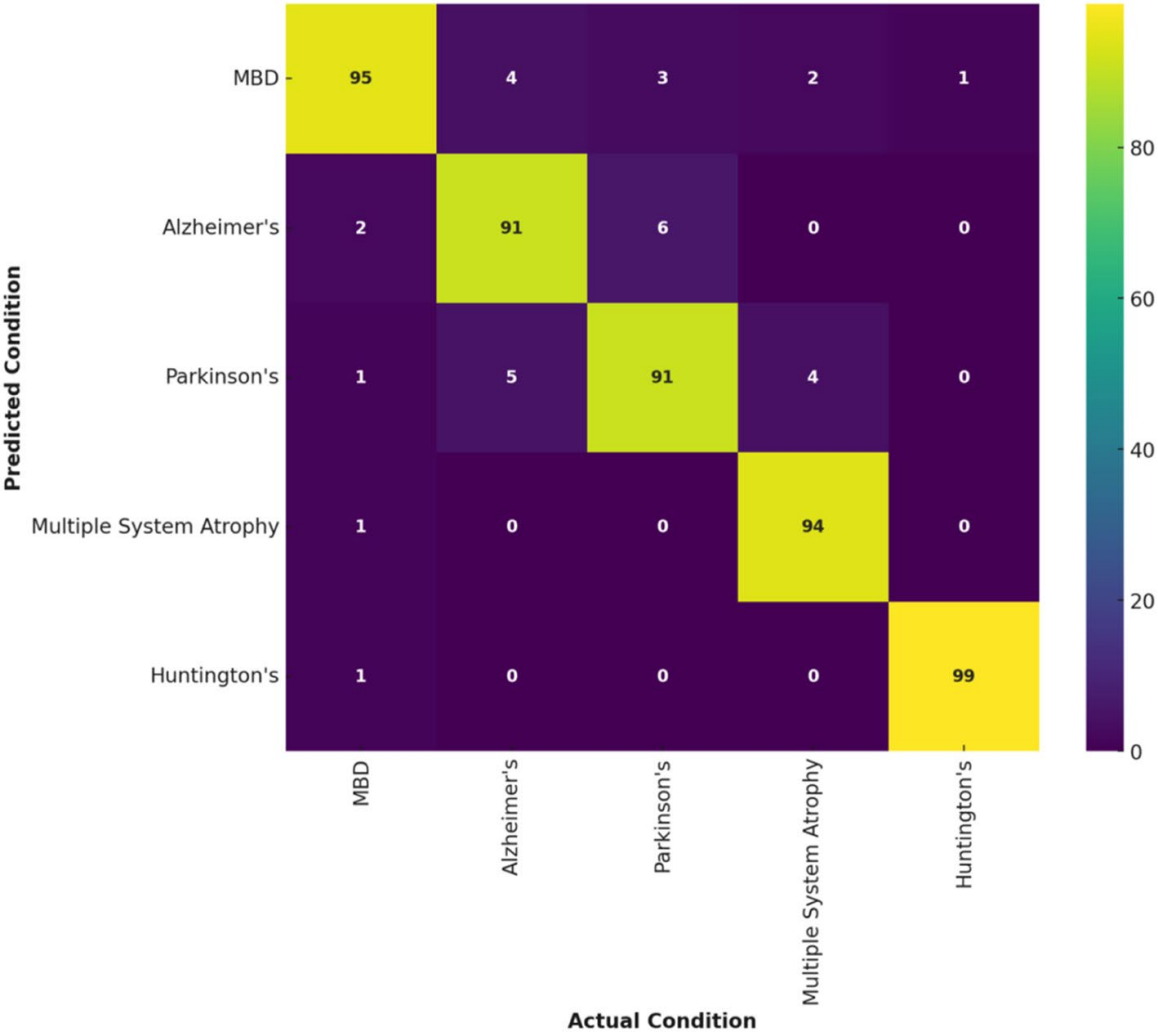
Confusion matrix of VAE + AM in detecting MBD

The confusion matrix shown in Fig. 4 demonstrates that the VAE + AM model achieves a remarkable accuracy of more than 90% in accurately detecting MBD. This is confirmed by 95 accurate predictions out of a total of 100. The VAE + AM model demonstrates a remarkable degree of accuracy, highlighting the effectiveness of its AM in conducting a detailed analysis of crucial visual elements relevant to MBD. The model has high specificity in differentiating MBD from other neurodegenerative illnesses such as Alzheimer’s, Parkinson’s, Multiple System Atrophy, and Huntington’s, as seen by the minimal number of misclassifications observed.

The advanced design of VAE + AM plays a crucial role in reaching such achievements. Incorporating the attention mechanism in the VAE framework enables the model to effectively prioritize and acquire knowledge from the essential characteristics in intricate medical pictures. It is critical for differentiating minor distinctions typical of different neurodegenerative disorders. The model’s capacity to provide a comprehensive and subtle depiction of the information in its latent space, together with its concentrated emphasis on crucial characteristics, improves its accuracy in diagnosis. VAE + AM demonstrate exceptional performance in accurately detecting MBD while effectively reducing both false negatives and false positives associated with comparable diseases. This makes it a powerful tool in medical diagnostics and image analysis.

**Fig. 5:**
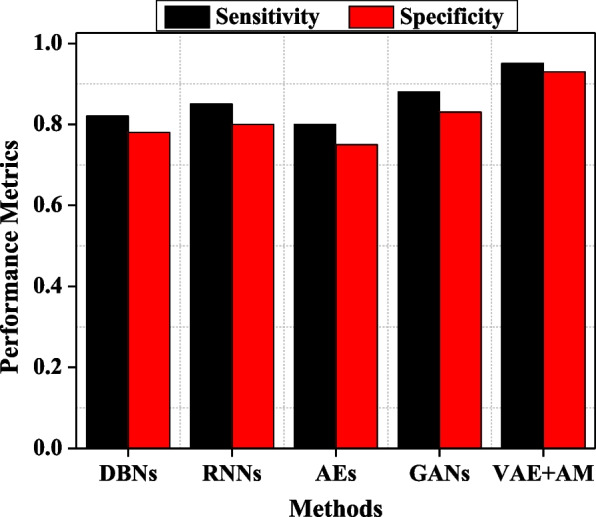
Sensitivity and specificity comparison among different methods

Figure 5 comprehensively analyses the sensitivity and specificity of five distinct techniques. Sensitivity quantifies the accuracy of adequately identifying genuine positive cases (MBD). A greater sensitivity implies superior accuracy in diagnosing MBD. The VAE + AM model has a sensitivity of 0.95, indicating its ability to correctly detect 95% of MBD patients. The heightened sensitivity may be attributed to the attention mechanism’s capacity to concentrate on the most relevant characteristics in MRI scans for MBD. Alternative techniques such as GANs, RNNs, and DBNs have less sensitivity, suggesting that they may fail to detect some MBD instances or need help to capture the subtle characteristics of the illness accurately.

Specificity measures the ability of a technique to accurately identify instances that do not have the illness being studied, known as non-MBD conditions. A high specificity indicates that the model effectively identifies instances that are not MBD, reducing false positives. The VAE + AM achieve a specificity score of 0.93, which suggests that it accurately detects 93% of non-MBD patients. This high level of reliability significantly reduces the likelihood of misdiagnosis. Although other approaches are successful, they could be better in comparison, which may result in increased false positives. Therefore, the outstanding outcome of VAE + AM in both criteria demonstrates its effectiveness.

**Fig. 6:**
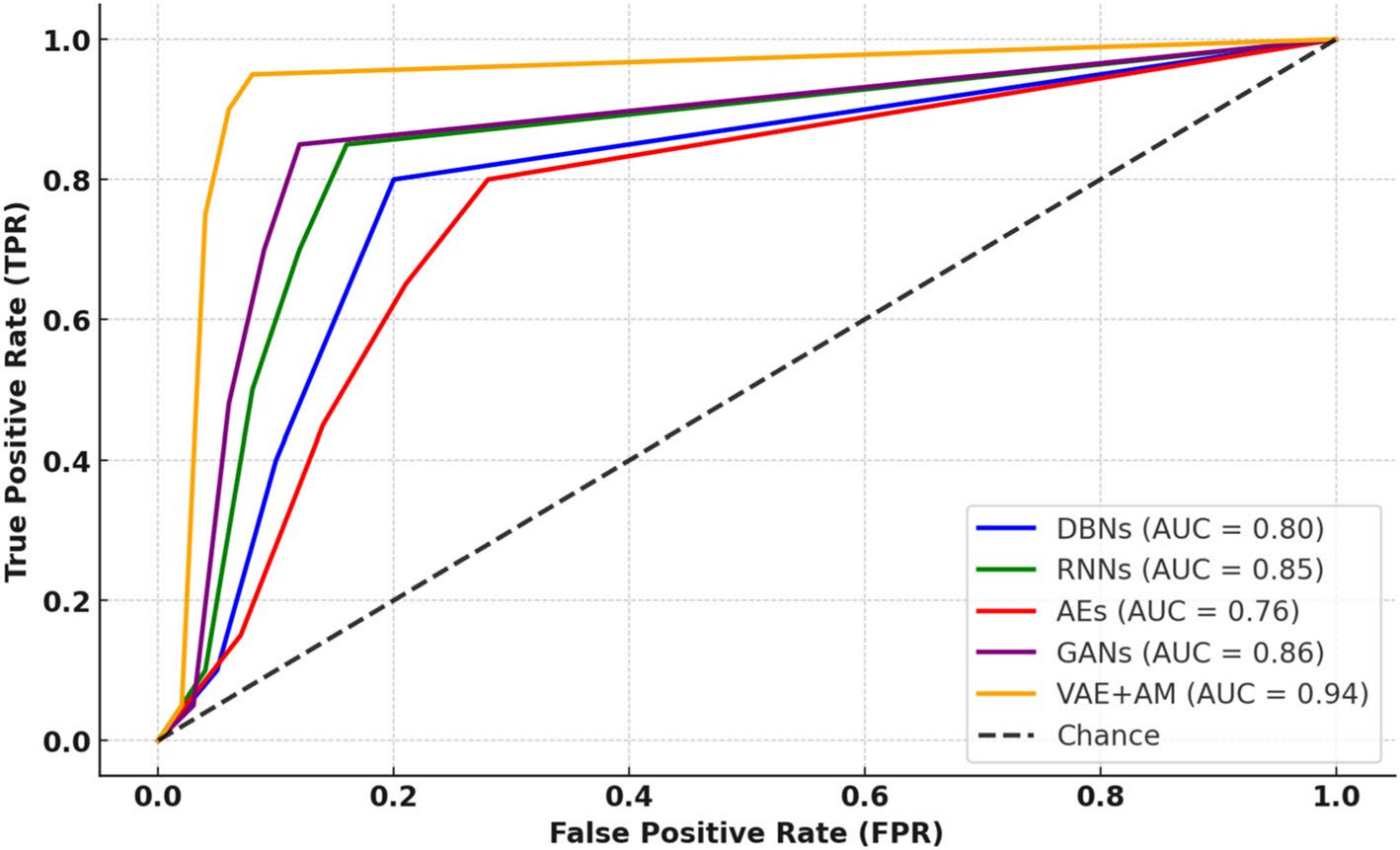
AUC-ROC comparison among different methodologies

Figure 6 depicts the ROC curve, which illustrates the diagnostic efficacy of several computational models in discriminating between patients with MBD. The actual positive rate (TPR) at different threshold levels is shown versus the false positive rate (FPR) on the curve. The VAE + AM model, shown by the line closest to the top left corner of the graph, demonstrates a better balance between sensitivity (TPR) and specificity (1-FPR), as seen by its higher AUC score of 0.94. The score achieved by the attention mechanism inside the VAE framework for this particular medical diagnostic job is much higher than that achieved by DBNs, RNNs, AEs, and GANs. This highlights the efficiency of the attention mechanism in improving the model’s accuracy. The VAE + AM model’s excellent performance in accurately categorizing MBD cases and eliminating false alarms is evident from its closeness to the ideal point (TPR = 1, FPR = 0).

**Fig. 7:**
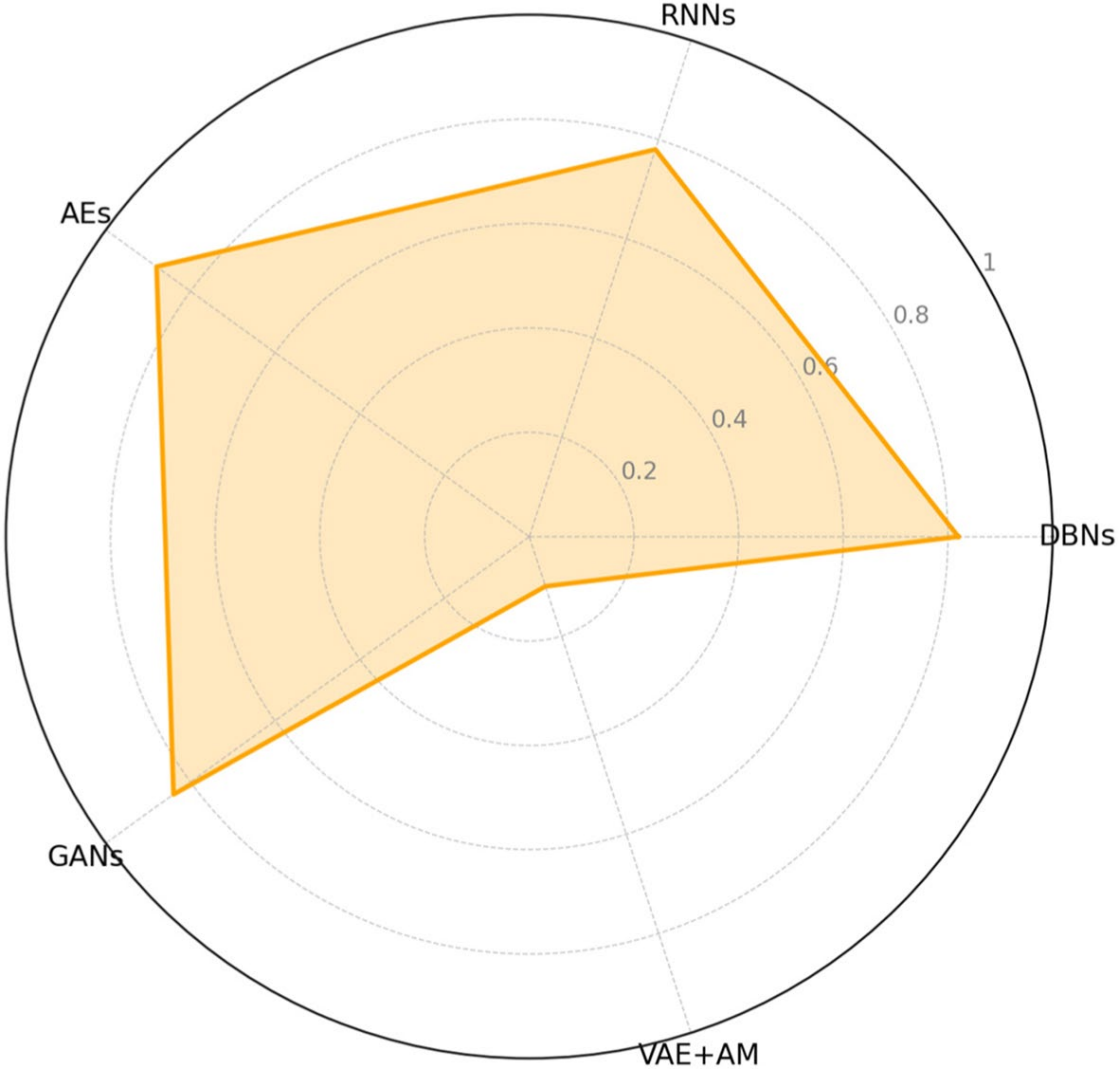
MSE evaluation for MBD detection

Figure 7 demonstrates that the combination of VAE + AM is successful in collecting the complexity of the data and efficient in its learning process. This leads to a consistent and considerable decrease in prediction errors, surpassing other approaches in identifying MBD. Based on the picture, it is evident that the VAE + AM approach is positioned closest to the centre point on its axis, suggesting that it has the lowest MSE among the studied methods. A shorter distance from the centre indicates a reduced margin of error, therefore indicating better performance. This implies that VAE + AM exhibits reduced prediction errors and superior generalization capabilities when evaluated on unfamiliar data. The superior performance of VAE + AM may be ascribed to many factors:

- ▪ The VAE + AM model incorporates an AM that strategically focuses on the most informative aspects of the input data, similar to how a human expert might concentrate on the most prominent elements. By using this focused methodology, a more refined model is developed that effectively captures the fundamental attributes of the data, leading to a reduction in Mean Squared Error (MSE).
- ▪ The VAE component of VAE + AM utilizes variational inference, which introduces a regularization impact by including the KL divergence term into the loss function. This promotes the model to acquire a well-organized latent space that exhibits greater generalization to unfamiliar data, reducing overfitting and decreasing mean squared error (MSE).
- ▪ The integration of variational autoencoding and attention in VAE + AM enables the extraction and use of resilient features highly suggestive of the fundamental patterns in the data. This is incredibly potent in intricate sectors such as medical diagnostics, where minute pattern variations are crucial for precise forecasts.
- ▪ The VAE + AM model’s ability to generate new data enables it to rebuild input using the latent representations it learned. This iterative process of improving reconstructions leads to a constant drop in MSE throughout training epochs, enhancing the model’s prediction accuracy.

The outcome of Table 6 shows the reciprocal KL divergence for each approach. The result clearly emphasizes the effectiveness of each model by measuring the degree of similarity between the model’s learning probability distribution (*Q*) and the actual data distribution (*P*). Lower KL divergence values suggest more similarity across *Q* and *P*, which is desired for improving model accuracy.The VAE + AM approach is distinguished by its minimal KL divergence (0.10), suggesting that it outperforms the other models in performance. The VAE + AM’s exceptional performance can be ascribed to its capacity to comprehend the MBD data’s intricate layout effectively. The AM of the model allows it to concentrate on essential aspects. At the same time, its variational component applies a regularization that helps the model achieve a more precise and comprehensive depiction of the data. In medical diagnostic jobs, accuracy is of utmost importance. The VAE + AM architecture is well-suited to fulfil these requirements, as seen by its superior KL divergence score.

**Table 6:** KL divergence values

**Methodology**	**KL Divergence (ϕ)**
DBNs	0.25
RNNs	0.20
AEs	0.30
GANs	0.18
VAE + AM	0.10

**Fig. 8:**
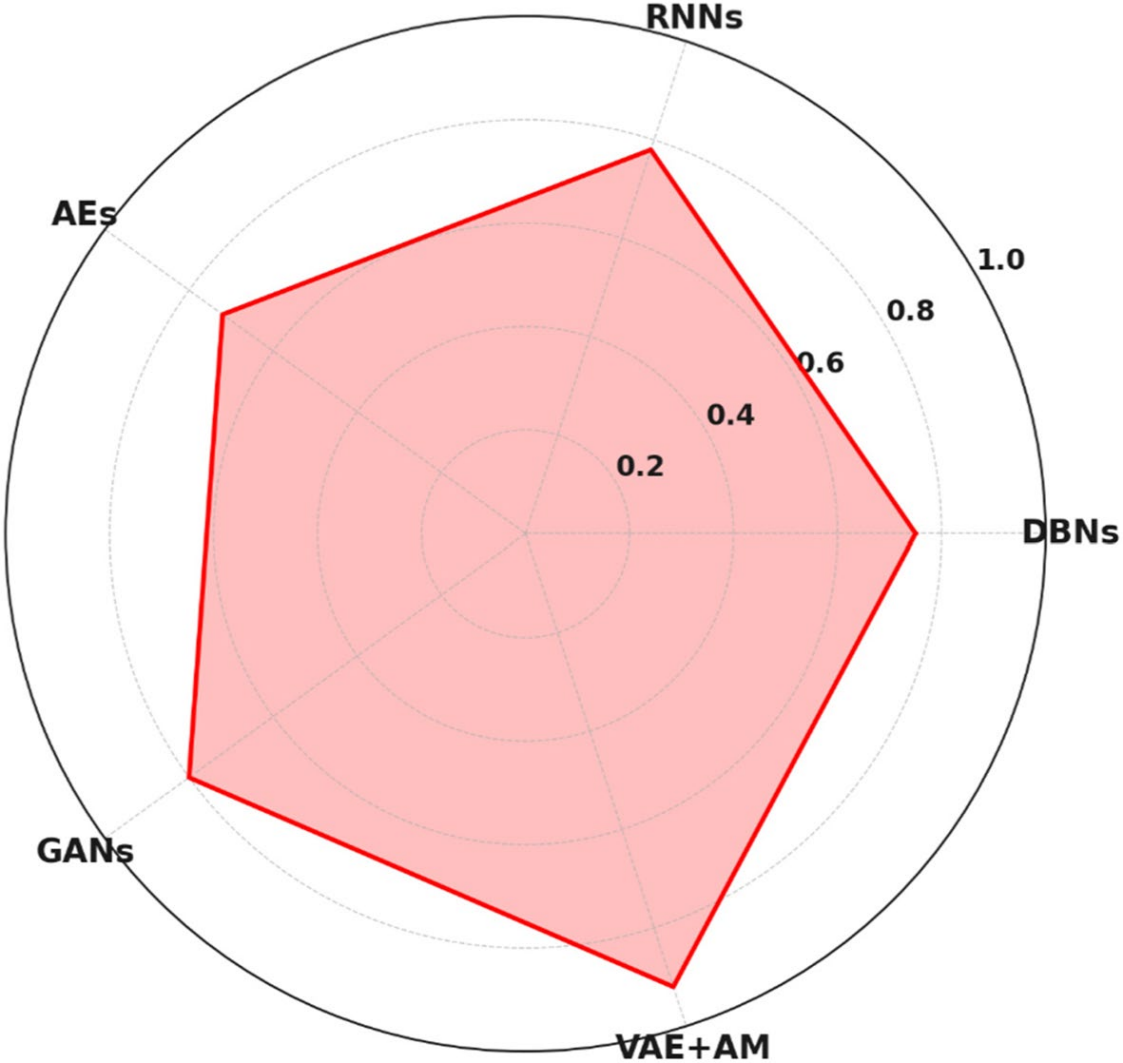
DCS for MBD detection across different methodologies

Figure 8 illustrates the DSC results for several approaches used to detect MBD. In this scenario, the DSC quantifies the resemblance between two sets, namely the projected pixel segmentation (u) and the ground truth pixel segmentation (v). The DSC is a numerical measure from 0 to 1. A DSC value of 1 shows a complete overlap between the anticipated and genuine segmentations, whereas a value of 0 signifies no overlap.

The distance from the centre to a point on each axe indicates the DSC value associated with that particular approach. The VAE + AM approach is shown as achieving the most significant distance towards the outside boundary of the chart, indicating a greater DSC and, hence, better results in the segmentation process of MBD identification. The model’s exceptional performance is probably attributed to the attention mechanisms included in the VAE framework. These mechanisms enable the model to concentrate on the most relevant areas of the picture, leading to precise segmentation and, therefore, a higher DSC.

The other techniques, namely DBNs, RNNs, AEs, and GANs, exhibit shorter radial distances. These distances correspond to lower DSC values, implying less accurate segmentation performance. The radar map successfully demonstrates the comparative study of segmentation accuracy among various models, clearly emphasizing the better accuracy of VAE + AM in correctly recognizing the regions of interest within the medical pictures for MBD detection.

**Fig. 9:**
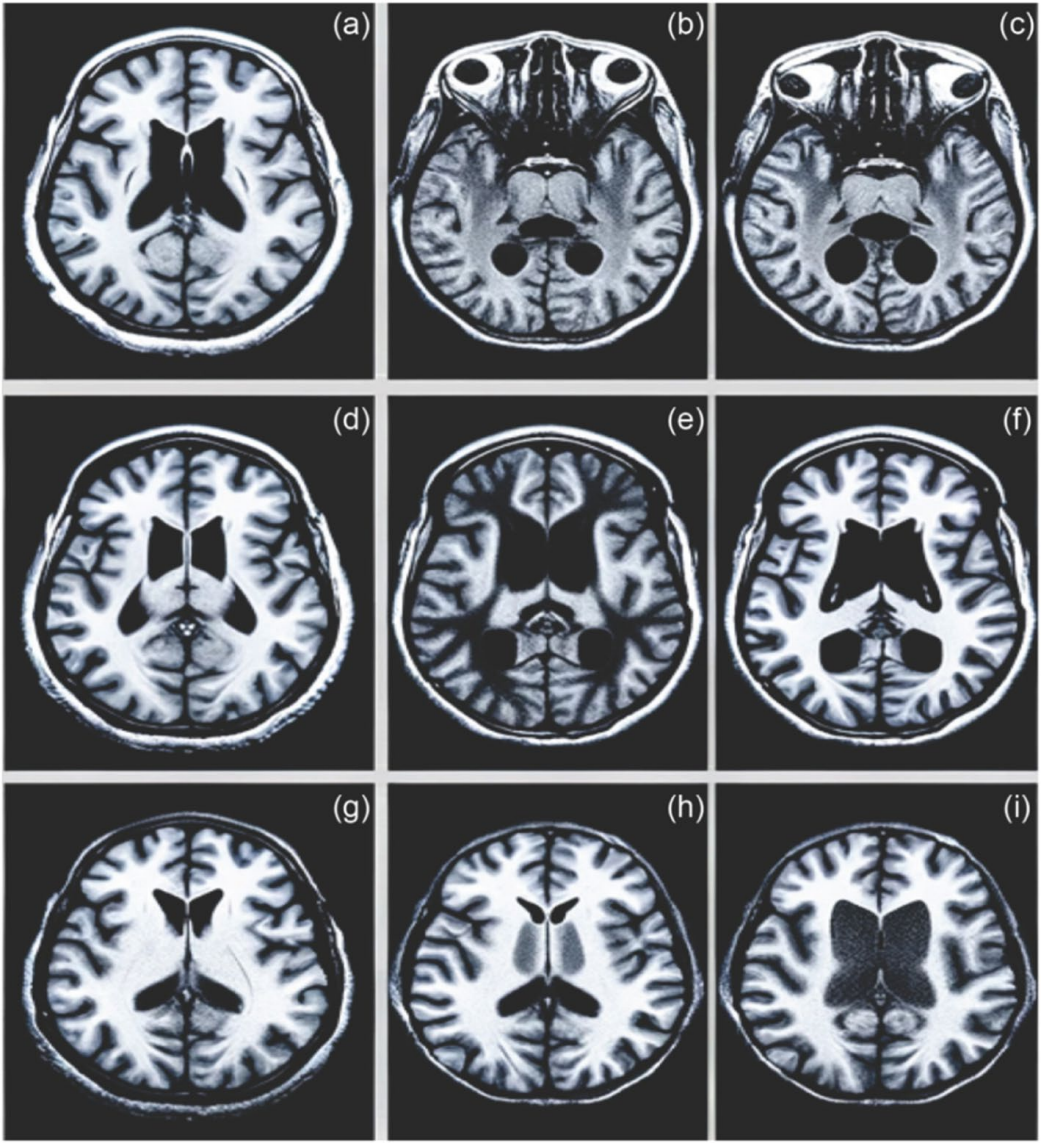
MRI outcome of VAE showing MBD phases, illustrating and highlighting key features using an attention mechanism

Concerning Fig. 2, a few sample visuals from (*a*) to (*i*) in Fig. 9 (the training outcomes of VAE) demonstrate the typical characteristics of MBD. Those features include hyperintensities and hypointensities in particular brain areas, showing effective extraction of these features.

The VAE, in conjunction with an attention mechanism, aims to acquire and emphasize significant characteristics within the data. The distinct delimitation of affected regions implies that the model has successfully detected and concentrated on crucial areas, hence improving the model’s diagnostic skills.

The attention mechanism in the VAE assigned more importance to the pertinent anatomical components, such as the corpus callosum, which is critical in detecting MBD. The diversity in both intensity and structure seen in the corpus callosum throughout the sample visuals demonstrates the assigned importance of the pertinent anatomical components by the attention mechanism in the VAE.

The images illustrate several manifestations of the corpus callosum’s form, ranging from atrophy (*d*) to alterations in signal intensity (*g* to *i*), which are indicative of the disease’s advancement. This suggests that the attention strategy successfully guided the VAE in reconstructing these crucial aspects.

The VAE has accurately reproduced the corpus callosum structure, displaying varied levels of intricacy that correspond to various phases and severity levels of MBD. The reconstructions indicate that the VAE has acquired a sophisticated, nonlinear correlation between the illness indicators, demonstrating the successful results of the training.

The implications of our research on utilizing deep learning techniques for detecting MBD are significant, particularly in enhancing diagnostic accuracy and efficiency in clinical settings. By integrating advanced deep learning models such as VAEs with attention mechanisms, our study offers a sophisticated approach that improves the sensitivity and specificity of MBD detection. This allows for earlier and more accurate disease identification, crucial for timely intervention and treatment planning. Furthermore, the methodology developed can serve as a blueprint for applying similar techniques to other complex neurological disorders, potentially transforming diagnostic processes across various medical conditions. These diagnostic tools’ increased reliability and precision also promise to reduce the overall healthcare burden by enabling more effective management and treatment of patients with MBD, ultimately leading to better patient outcomes and resource utilization.

## Conclusion and future directions

Integrating sophisticated deep learning techniques in medical diagnostics has significantly enhanced the identification of intricate and uncommon neurological illnesses like MBD. The combination of VAEs with AM has shown significant efficacy in tackling the difficulties presented by MBD, a disorder characterized by inconspicuous and sometimes disregarded radiological indications. Incorporating attention processes in VAEs in this work has resulted in a substantial improvement in diagnostic accuracy, shown by achieving an accuracy rate above 90%, a sensitivity score of 95%, and a specificity of 93%, as indicated in the model assessments. Furthermore, the model’s DSC of 0.92 demonstrates outstanding proficiency in accurately identifying and delineating damaged regions in MRI brain scans. The numbers illustrate the model’s capacity to accurately identify MBD and emphasize its potential to minimize false positives, making it a reliable tool for healthcare professionals. As a result, this sophisticated method makes it easier to identify MBD early and with precision. This leads to the development of more personalized and efficient treatment strategies, highlighting the significant influence of VAEs with AM on the practice of diagnosing neurodegenerative diseases.

We intended to include several types of data, such as genetic and clinical information with MRIs, to enhance the precision of the diagnostic model. In addition, the model might be improved by investigating transfer and federated learning methods. Such techniques let the model adjust to various patient groups and real-world clinical environments, improving its generalization capacity.

## Data Availability

The data that support the findings of this study are openly available in OpenNeuro at https://openneuro.org/datasets/ds004392/versions/1.0.0/download, https://github.com/OpenNeuroDatasets/ds004392.git, Accession number: ds004392.
